# Enhanced Antihypertensive Activity of Candesartan Cilexetil Nanosuspension: Formulation, Characterization and Pharmacodynamic Study

**DOI:** 10.3797/scipharm.1103-17

**Published:** 2011-07-05

**Authors:** Chetan Detroja, Sandip Chavhan, Krutika Sawant

**Affiliations:** Drug Delivery Laboratory, Centre of Relevance and Excellence in NDDS, G.H. Patel Building of Pharmacy, Pharmacy Department, Faculty of Technology and Engineering, The Maharaja Sayajirao University of Baroda, Vadodara-390002, India

**Keywords:** Candesartan, Saturation solubility, Nanosuspension, Bioavailability, DOCA salt model

## Abstract

The objective of the present investigation was to enhance the oral bioavailability of practically insoluble Candesartan cilexetil [CC] by preparing nanosuspension. The nanosuspension was prepared by media milling using zirconium oxide beads and converted to solid state by spray drying. The spray dried nanosuspension of CC [SDCN] was evaluated for particle size, zeta potential, saturation solubility, crystallanity, surface morphology and dissolution behavior. SDCN showed particle size of 223.5±5.4 nm and zeta potential of −32.2±0.6 mV while saturation solubility of bulk CC and SDCN were 125±6.9 μg/ml and 2805±29.5 μg/ml respectively, showing more than 20 times increase in solubility. Differential Scanning Calorimetry [DSC] and X-ray diffraction [XRD] analysis showed that crystalline state of CC remained unchanged in SDCN. Dissolution studies in phosphate buffer pH 6.5 containing 0.7% Tween 20 showed that 53±5% of bulk drug dissolved in 15 min whereas SDCN was almost completely dissolved exhibiting higher dissolution velocity and solubility. Transmission electron microscopy [TEM] revealed that nanocrystals were not of uniform size, and approximately of oval shape. Pharmacodynamic study based on deoxycorticosterone acetate [DOCA] salt model was performed in rats to evaluate *in-vivo* performance, which showed 26.75±0.33% decrease in systolic blood pressure for nanosuspension while plain drug suspension showed 16.0±0.38% reduction, indicating that increase in dissolution velocity and saturation solubility leads to enhancement of bioavailability of SDCN when compared to bulk CC suspension. Thus, the results conclusively demonstrated a significant enhancement in antihypertensive activity of candesartan when formulated as nanosuspension.

## Introduction

**Fig. 1.**
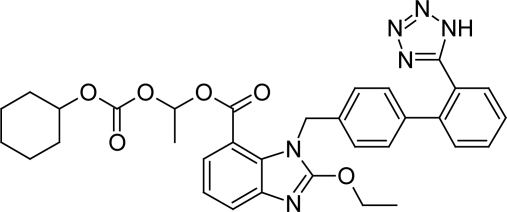
Chemical Structure of Candesartan Cilexetil

Candesartan, (±)1-{[(cyclohexyloxy)carbonyl]oxy}ethyl 2-ethoxy-1-{[2′-(1*H*-tetrazol-5-yl)-biphenyl-4-yl]methyl}-1*H*-benzimidazole-7-carboxylate, a nonpeptide, is a selective AT1 subtype angiotensin II receptor antagonist indicated for the treatment of hypertension alone or in combination with other antihypertensive agents [1, 2]. Candesartan cilexetil [CC], Fig.1, is a prodrug that is hydrolyzed to candesartan during absorption from the gastrointestinal tract. CC has log P value of 6.1 and the aqueous solubility of CC is less than 5×10^−5^ g/L which may be the reason for very low bioavailability i.e. about 15% [3, 4]. Solubility of poorly soluble compounds can be increased by formulating as aqueous pH-shifted solutions, provided the molecules are ionizable, in mixtures of water and organic co-solvents, or by solubilization in Cyclodextrins [5, 6] or using emulsions [7, 8]. With the exception of the pH-shifted aqueous solutions, significant amounts of additives are often needed to increase the solubility into the millimolar range, which may induce unwanted side effects [9]. It would be more desirable to have a universal formulation approach to process any poorly soluble drug. A classical formulation approach for such poorly soluble drugs is micronization, where a coarse drug powder is milled to an ultrafine powder with a mean particle size being typically in the range of 1–10 μm. The principle is to increase the dissolution velocity by enlarging the surface area of the drug powder [10]. However, many of the new drugs exhibit so low solubility, that even micronization does not help to improve their bioavailability. Similarly, micronization of CC did not lead to significant enhancement in dissolution rate [11]. Thus, there is urgent need to have some innovative formulation approach to enhance the bioavailability.

Nanosuspension [NS] is the best alternative and universal approach for poorly soluble drugs, especially which are insoluble in aqueous as well as organic media simultaneously, to enhance bioavailability [12–14]. Advances in technology and better understanding of the NS systems have attracted attention of formulation development scientists towards preparation of such systems. NS is sub-micron colloidal dispersion of drug particles which are stabilized by surfactant. It has two outstanding features: it is able to increase saturation solubility and consequently also increases the dissolution velocity [15, 16].

In general, NS can be produced by three techniques, top-down, bottom-up and combination of both. Media milling and high pressure homogenization are two basic approaches of top-down technique. In milling approach, drug powder is dispersed in a surfactant solution and the obtained suspension is poured in the milling container containing milling beads made from glass, stainless steel, zirconium oxide, or highly cross-linked polystyrene resins. Then drug is nanosized by moving the beads, either by using a stirrer or by moving the mill container itself [17]. The Media milling techniques is very easy and simple method to prepare NS.

Although the bottom-up approaches hold tremendous potential with respect to improving bioavailability by obtaining smaller particle sizes [<100 nm] and amorphous drug particles, no commercial application of these systems has yet been realized. Recently, it was reported that amorphous NSs are prone to Ostwald ripening; a process that leads to the growth of particles as time progresses. However, by incorporating a second component of extremely low aqueous solubility [inhibitors], Ostwald ripening can be inhibited. But, to inhibit ripening, the drug/inhibitor mixture [in the particles] must form a single phase; that is, the drug must be soluble in the inhibitor. However, when phase separation between the drug and inhibitor occurs, Ostwald ripening cannot be prevented [18]. Also, preparation of stable amorphous NS is tough and long term stabilization of amorphous drug particles is not easy. Hence, the easier and more viable top-down approaches are being preferred for the production of NS.

Currently, nine NS based formulation are available in the US market and all are FDA approved from the year 2000 on. All these products are based on top-down approaches, eight relying on media milling and one on high-pressure homogenization. This itself explains the popularity of the top-down approach and also emphasizes the ease of industrial scale up of this technology. A third remarkable point is that all commercial products are intended for oral delivery [19, 20].

Benefits of these systems are proved by number of products in market based on these techniques. Hence, the aim of present investigation was to prepare NS of CC for the enhancement of oral bioavailability. The drug [CC] was nanosized by media milling technique, characterized, and evaluated for improvement in bioavailability by pharmacodynamic study by measuring the blood pressure lowering effects in deoxycorticosterone acetate [DOCA] salt model induced hypertensive rats.

## Material and Methods

### Materials

Candesartan cilexetil [CC] was obtained as gift sample from Alembic Research Centre, Baroda, India. Zirconium oxide beads were kindly provided by SPARC, Baroda, India. Poloxamer 407[Pluronic F 127] was purchased from BASF, Germany. Polyvinlylpyrollidone [PVP K-30], methanol and Tween 20 [polyoxyethylene sorbitol laureate] were purchased from S.D. Fine Chemicals, India. Water was filtered through Millipore 0.22μm filter before used.

### Preparation of candesartan cilexetil Nanosuspension

NS was prepared by media milling technique using zirconium oxide beads as milling media [diameter ranging from 0.4 mm to 0.7 mm] [21]. In 20 ml glass vial, 6 g of zirconium oxide beads and 5 ml water were added. Then 50 mg Poloxamer 407 and 25 mg drug were incorporated and comminution was carried out on magnetic stirrer [Remi Equipment Pvt Ltd, India] at 500rpm for 14 hrs [9]. Then, Mannitol at a concentration of 5%w/v of total formulation was dissolved in the resultant NS. This suspension was spray dried to get dried powder using LU-227 Advanced Spray Dryer [Lab Ultima, Mumbai, India]. Operation parameters in spray dryer were: inlet temperature: 115°C, outlet temperature: 55°C, aspiration: 1200 rpm and feed pump: 3rpm [22]. Plain drug suspension was prepared by dispersing the micronized powder and Poloxamer in water to get suspension with respective concentration of drug and surfactant as that of nanosuspension.

### Characterization of Nanosuspension

#### Particle size

Mean particle size and size distribution [polydispersity index] of the prepared NS was determined by using Malvern Zetasizer [NanoSeries Nano-ZS, Malvern Instruments, UK] which follows principle of light diffraction, also called Photon correlation spectroscopy [PCS]. Prior to the measurement, the samples were appropriately diluted with water to a suitable scattering intensity and re-dispersed by shaking before measurement.

#### Zeta potential

The Zeta potential is a measure of the electric charge at the surface of the particles, indicating the physical stability of colloidal systems. The zeta potential values higher than |30mV| indicate long-term electrostatic stability of aqueous dispersions [16]. In this study, the Zeta Potential was assessed by determining the electrophoretic mobility of the particles using Malvern Zetasizer [NanoSeries, Nano-ZS, Malvern Instruments, UK].

#### Saturation solubility

Saturation solubility is defined as being a compound-specific constant depending only on the temperature and polymorphic state. However, apart from these, saturation solubility is also a function of the particle size. This size-dependency comes only into effect for particles having a size below approximately 1 μm [16]. For determination of saturation solubility, prepared nanosuspension was filled in a vial and kept for 24 hrs stirring to ensure saturation. Then 1.5 ml of NS was filled in 2 ml centrifugation tube and centrifuged at 25000 rpm for 30 minutes[3K 30 Sigma laboratory centrifuge, Osterode, GmBH]. Supernatant was filtered through 0.2 μm filter paper and analyzed spectrophotometrically using UV-visible spectrophotometer [UV-1700, Shimadzu AS, Japan] at 259 nm after suitable dilution with phosphate buffer [pH 6.5] containing 0.7% Tween 20, which was used as blank. For determination of saturation solubility of bulk drug, excess quantity of bulk CC was added to 1.5 ml of water and then saturation solubility was also measured in the same way.

#### X-ray Diffraction [XRD]

XRD studies were performed to study effect of milling and spray drying on the crystallinity of CC. The XRD studies of bulk CC powder, Poloxamer 407, mannitol and SDCN were carried out using X-ray diffractometer [XRD] [Brucker AXS, model D8 advanced, Germany] with Cu line as source of radiation at Electrical Research and Development Agency, Baroda, Gujarat, India. Standard runs were taken using 40kV voltage, 40mA current and scanning rate of 0.02°/min over a 2θ range of 5–50°.

#### Differential Scanning Calorimetry [DSC]

DSC studies were performed to investigate the effect of surfactants, milling process and drying process on the inner structure of candesartan cilexetil and to confirm crystallinity result obtained by XRD. The DSC thermograms of bulk CC powder, Poloxamer 407, their physical mixture and SDCN were taken on a DSC-60 Shimadzu [Shimadzu AS, Japan] Differential Scanning Calorimeter between 40–300 °C at a heating rate of 10 °C/min with nitrogen supplied at 40 ml/min.

#### Transmission Electron Microscopy [TEM]

Morphological evaluation of NS was conducted by Transmission Electron Microscopy. TEM images were recorded using Transmission Electron Microscope [Philips Technai-20]. The liquid NS formulation was dropped on copper-gold carbon coated grid and allowed to dry. This grid was then mounted in the instrument and photographs were taken at various magnifications.

#### Dissolution studies

Dissolution experiments were performed using USP 24 paddle instrument [Electrolab TDT-06P]. The dissolution medium was used in this study i.e. phosphate buffer [pH 6.5] containing 0.7% Tween 20 [23]. The medium (250 ml) was gently transferred into the dissolution vessel so as to minimize foaming of the medium during the experiment. Dissolution was performed at 37°C, using a paddle speed of 100 rpm. Samples of bulk CC and SDCN equivalent to 15 mg of CC were added to dissolution vessels and aliquots were withdrawn at suitable time intervals for 60 min, and filtered immediately through 0.1μm PTFE syringe filter [Whatman Inc., Clifton, NJ, USA] [21]. Subsequently, same volume of fresh medium was added to the dissolution vessel. Quantification of the samples was done by UV analysis at 259nm. The experiment was performed three times.

### Stability studies

Stability studies of the NS and SDCN were conducted at two different storage conditions [Room temperature (RT) and Refrigerated (RF i.e. 2–8 °C)] for 2 months. Three batches for both formulations were used for each condition. The physical stability of nanosized formulations was judged in terms of particle size determined at specific time intervals. Assay was also carried out periodically to determine the chemical stability of CC. To determine the drug content, formulation with amount equivalent to 2.5 mg of CC was dissolved in methanol, diluted appropriately, filtered and absorbance of resulting solution was measured spectroscopically at 255.5 nm.

### In vivo studies

A pharmacodynamic method was applied to determine enhancement in bioavailability due to NS of CC as compared to plain drug suspension. Candesartan inhibits the pressor effects of angiotensin II infusion in a dose-dependent manner [24,25]. Hence, decrease in pressor effect can be directly correlated with the amount of drug that reaches the systemic circulation i.e. to the bioavailability of the drug. In other words, greater the inhibition of the pressor effect, higher the bioavailability of the administered formulation. The pharmacodynamic study was thus based on this hypothesis.

DOCA [deoxycorticosterone acetate] salt model was applied to induce hypertension in rats [26]. After induction of hypertension, treatment was started with plain drug suspension and NS and blood pressure was measured by tail-cuff method using LE 5002 Storage Pressure Meter [Letica Scientific Instruments]. The animal experiments are conducted in full compliance with Institutional Animal Ethical Committee [IAEC] regulations, as per CPCSEA guidelines. The registration number of our institute is 404/01/a/CPCSEA.

#### DOCA salt hypertensive rats

Female Wistar rats [weight approximately 200–250 g] obtained from Zydus Research Centre, Ahmadabad, India were used for the study. These animals were divided into six groups, each containing four rats. All rats were uninephrectomised under anesthesia with intraperitoneal ketamine [100 mg/kg]. Kidneys were visualized by a right lateral abdominal incision. The right kidney was removed after ligation of adjoining renal vasculature and ureter with sutures i.e. uninephrectomy. After one week recovery period, uninephrectomized rats were given either no further treatment [UNX rats] or 1% NaCl in drinking water with subcutaneous injections of deoxycorticosterone acetate [DOCA; 25mg in corn oil every fourth day] [DOCA-salt rats] [25]. DOCA-salt rats were further sub-grouped into five according to treatment given to them: D1 & D2-low dose nanosuspension and plain drug suspension, respectively [0.5mg/kg/day], D3 & D4-high dose nano-suspension and plain drug suspension, respectively [5mg/kg/day], D0-no treatment [DOCA control] [27]. To get bulk drug suspension, plain CC with equivalent quantity of surfactant of NS was suspended into distilled water before administration, while nanosuspension was administered as such after suitable dilution. After 14 days, all subgroups of DOCA-salt rats except D0 subgroup were orally administered candesartan cilexetil formulations daily for further 7 days.

#### Measurement of systolic blood pressure

Systolic blood pressure was measured once a week before drug administration for first two weeks [using tail-cuff method]. During treatment, systolic blood pressure was measured daily for all subgroups of DOCA salt rats except D0, 2–3 hours after administration. In UNX rats and D0 rats, blood pressure was measured once a week throughout the experiment.

#### Statistical analysis

ANOVA was applied followed by t-test to determine differences in decrease in blood pressure between groups; p < 0.05 was considered significant.

## Results and Discussion

### Preparation of candesartan cilexetil Nanosuspension

Media milling technique using zirconium oxide beads was adopted as it uses least number of equipments and is a relatively easy method. By using factorial design the formulation parameters were optimized (Data not shown). The particle size of optimized batch was found to be 223.5±5.4, indicating the usefulness of media milling method for nanosizing. Effective milling was observed at bead concentration of 120% w/v and no further improvement was observed when concentration of beads was increased beyond this limit. Plain drug suspension particle size was determined by Malvern 2000 Mastersizer and found to be 54± 8.0 μm.

Stabilization of the nanoparticles in a NS form requires a stabilizer that binds onto the particle surface [28, 29]. Poloxamer adsorb strongly onto the surface of hydrophobic nanoparticles via their hydrophobic polyoxypropylene centre block and have been shown to be quite successful in regard to stabilization of nanoparticles [30]. Two stabilizers, Poloxamer 407 and PVP K-30 were tried to stabilize the formulation. The average particle size obtained using Poloxamer 407 was 223.5±5.4 nm while with PVP K-30, it was 236.0 ± 14.0 nm. The PDI values were also less for Poloxamer 407. Based on these results, Poloxamer 407 was found to be a better stabilizer of the NS of CC.

To incorporate nanocrystals into solid dosage forms [e.g. tablets, capsules, pellets etc.], the transformation of the liquid aqueous nanocrystals into a dry nanocrystals powder is necessary. Technically, it can be achieved using established unit operations such as freeze-drying, spray-drying, pelletization or granulation [31]. But, choice of the drying technology is important, because it needs to be ensured that the nanocrystals can be re-dispersed as separated particles and do not aggregate, which would lead to a loss of their special properties. Spray drying is a commonly used method to transform a crystalline drug substance into its high energy, thermodynamically metastable, amorphous state. It is simple, widely used and effective for nanosizing the drug.

In the present investigation, spray drying was performed to dry the aqueous NS of CC. In spray drying, a matrix former like sugar is often added to the suspension prior to the drying operation to preserve particle size of nanocrystals. Typical matrix formers added prior to drying are water soluble sugars like mannitol, dextrose, sucrose. In this study, mannitol at a concentration of 5%w/v of total formulation was added. SDCN was further investigated with respect to redispersability and particle size. SDCN could be easily and completely redispersed upon the addition of water without aggregates or agglomerates.

## Characterization of Nanosuspension

### Particle size analysis

The particle size [PCS] of the NS was determined before and after spray drying to study the effect of spray drying on particle size. PCS before spray drying was 223.5±5.4 nm with a polydispersity index [PI] of 0.234±0.021 while after re-dispersion of SDCN, the PCS and PI were 229.6±4.0 nm and 0.237±0.018, respectively. This data reveals that converting the NS into the dry powder using spray drying had no adverse influence on the particle size, polydispersity index and redispersability of CC nanocrystals.

#### Zeta potential

Colloidal systems must preserve their characteristics like particle size and colloidal stability to get advantage of these systems. Technically, colloidal stability is measured in the form of zeta potential. Generally, zeta potential value of ±30 mV is the minimum for a physically stable NS solely stabilized by electrostatic repulsion and the corresponding value is about ±20 mV in the case of a combined electrostatic and steric stabilization [16]. Zeta potential of NS and SDCN was found to be −33.2±0.86 mV and −32.2±0.59 mV. Thus, it was concluded that the systems had sufficient stability.

#### Saturation solubility

Saturation solubility will increase if the particle size of the nanocrystals is reduced below particular limit. Thus, nanonization might leads to increase in the saturation solubility. Buckton and Beezer assumed that the enhancement of solubility is valid only for sparingly soluble particles of less than 1 μm in size [32]. The average particle size of re-dispersed CC nanocrystals was below 1 μm, thus, theoretically it will help in improving saturation solubility and thus bioavailability. Saturation solubility was determined in the official dissolution medium for CC. Saturation solubility of SDCN and bulk CC at room temperature in phosphate buffer [pH 6.5] containing 0.7% Tween 20 was 2805±29.5 μg/mL and 125±6.9 μg/mL respectively. Thus, saturation solubility of SDCN was 22.44 times that of bulk drug. This greater than 200% increase in solubility of CC due to particle size reduction can be expected to enhance dissolution velocity and bioavailability, justifying the objective of this work. There was no significant difference in saturation solubility of drug before and after spray drying i.e. after addition of mannitol. Also in case of physical mixture (CC and poloxamer) no significant difference in saturation solubility was observed.

#### X-ray Diffraction pattern [XRD]

**Fig. 2.**
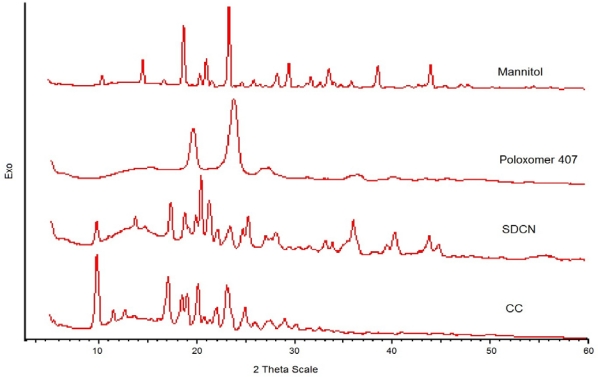
X-ray Diffraction pattern of bulk drug, formulation, Poloxamer 407 and Mannitol

XRD pattern of bulk CC, SDCN, Poloxamer 407 and mannitol are shown in Fig. 2. The major peaks of the CC are present in SDCN and the additional peaks appeared in SDCN are may be due to mannitol. These results showed that crystalline state of the bulk drug and SDCN was same indicating milling and drying processes did not induce a crystalline or polymorphic transition of the drug. Likewise, stabilizers did not influence the crystallinity of the candesartan cilexetil nanocrystals. In general, crystalline substances are physically more stable compared to amorphous forms. Therefore, it can be assumed that the spray dried nanocrystals will be physico-chemically stable during the storage. It can also be safely assumed that, better physicochemical properties such as observed; enhanced solubility and dissolution velocity can be attributed to the particle size reduction and not to alterations in crystalline state.

#### Differential Scanning Calorimetry [DSC]

**Fig. 3.**
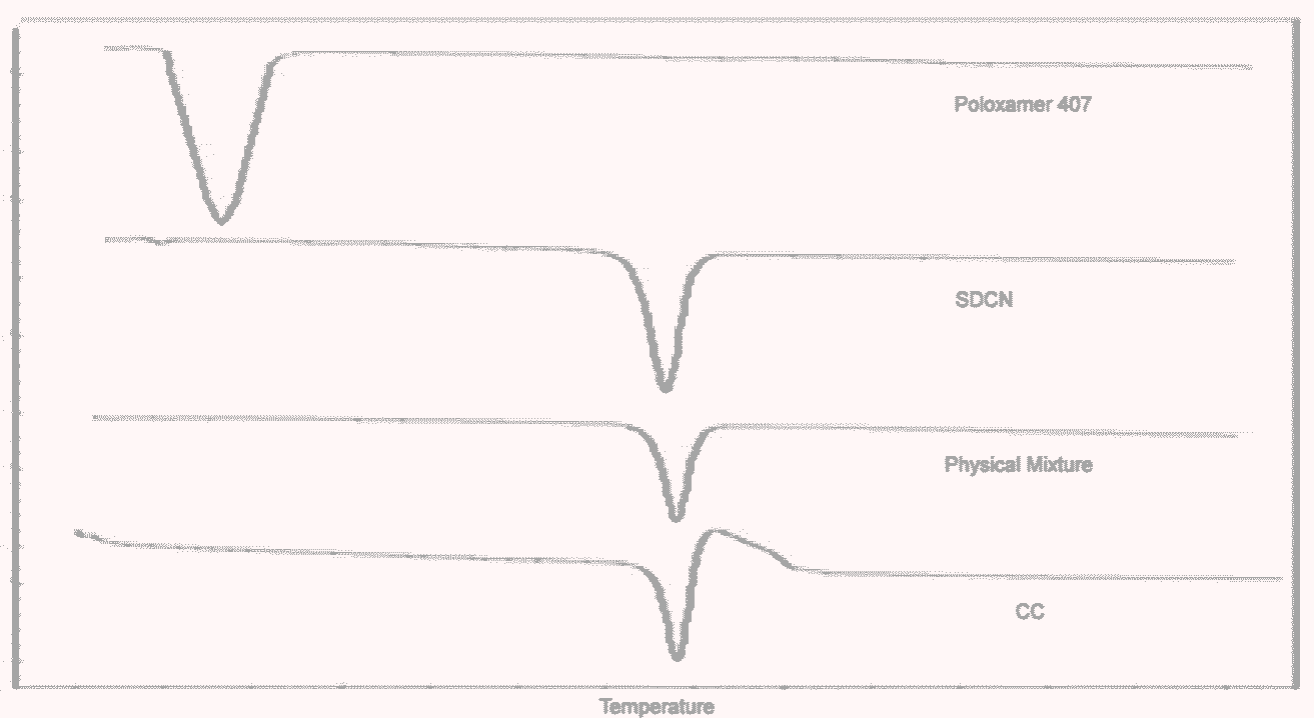
DSC thermogram of Candesartan cilexetil bulk powder, Poloxamer 407, physical mixture and spray dried nanosuspension(SDCN).

The DSC studies were performed to study the effect of nanosizing and spraying drying on solid state of CC. The DSC thermograms of CC bulk powder, SDCN, physical mixture and Poloxamer 407 are shown in Fig. 3. The DSC thermogram of bulk CC powder showed a sharp endothermic peak at 168°C followed by an exothermal peak at which is due to decomposition of the drug [33].The thermogram of Poloxamer showed endothermic peak at 55°C. Both, Physical mixture and the formulation showed sharp endothermic peaks at 168°C which drug remained in crystalline state. We can also conclude that the melting curve of CC was not influenced by either stabilizer or milling and drying process. Thus, the combined results from XRD and DSC studies show that nanosizing and spray drying does not affect the crystallinity of candesartan cilexetil.

#### Transmission Electron Microscopy [TEM]

**Fig. 4.**
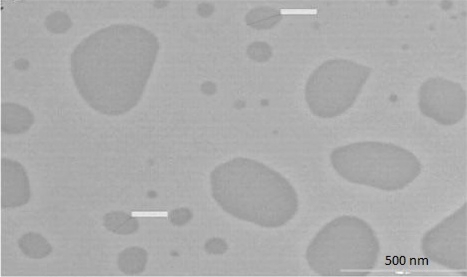
TEM photograph of Nanosuspension (Bar line= 500nm).

The morphological characteristics of the NS was determined using TEM [Fig. 4]. It was observed that the nanocrystals were not of uniform size and approximately of oval shape and the non-uniformity of the nanocrystals could be attributed to method used for nanosizing. It can also be seen that the drug particles were non-aggregated and uniformly dispersed in the medium. This was also reflected by comparative high polydispersivity index [PDI] of 0.234± 0.21.

#### Dissolution study

The most important feature of nanocrystals is the increase in the dissolution velocity, not only because of increase in surface area but also because of increase in saturation solubility. This phenomenon is best explained by Noyes-Whitney law [Eq. 1].

Eq. 1. $$\frac{\text{dm}}{\text{dt}}=\frac{\text{DA}}{h}\left[C_{s}-C_{x}\right]$$

Where, dm/dt= dissolution velocity, D = diffusion coefficient, A = surface area, C_s_ = saturation solubility, C_x_ = bulk concentration, and h = diffusional distance over which the concentration gradient occurs. Division of the equation by the volume v leads to dc/dt. It is obvious that an increase in A increases the dissolution velocity. According to Noyes-Whitney, the increase in C_s_ in addition to A further increases the dissolution velocity. According to the Prandtl equation [Eq. 2], the diffusional distance h decreases for very small particles.

Eq. 2. $$h_{H}=k\left[L^{1/2}-V^{2/3}\right]$$

Where h_H_= hydrodynamic boundary layer thickness; k= constant; L= length of surface in direction of flow, V= Relative velocity of flowing liquid against flat surface

**Fig. 5.**
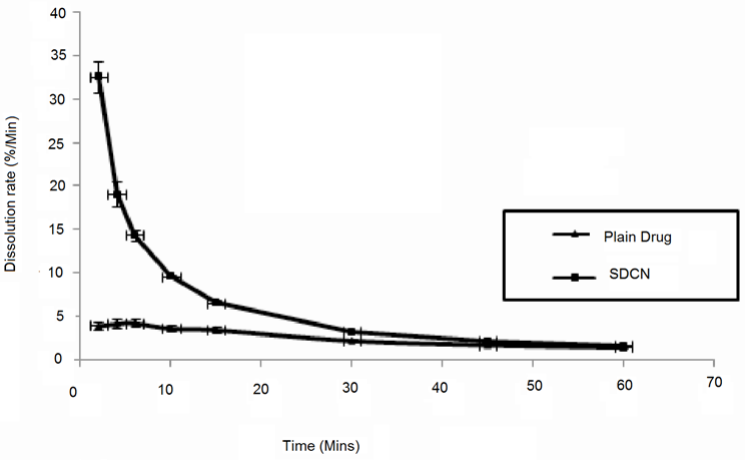
Dissolution rate of plain drug and spray dried nanosuspension of Candesartan cilexetil.

When the dissolution profile of the SDCN was compared with bulk CC, improved dissolution was observed. In SDCN, 76.3%±3.4 of the drug got dissolved within 4 minutes and about 99.4±3.6% within 15 minute, while bulk CC showed only 17.0±2.0% release at the end of 5 minutes and 53.5±5.0% release in 15 minutes. Thus, there was improvement in dissolution rate for SDCN compared to plain drug [Fig. 5]. The particle size reduction from 54± 8.0 μm to 223.5±5.4 is responsible for this improvement as the excipient in both case are same. This dissolution rate of the spray dried nanocrystals was distinctly superior compared to plain drug, which might be attributed to increase in surface area, saturation solubility and decrease in diffusional distance for nanocrystals, showing complete dissolution within minutes.

### Stability studies

#### Physical stability

**Fig. 6.**
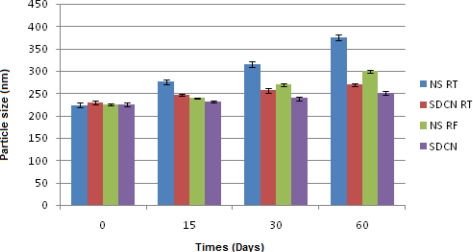
Change in particle size of nanosuspension and spray dried nanosuspension at various conditions.

Particle size of NS formulation was measured after 15, 30, and 60 days. The results are shown Fig. 6. Particle size of NS increased from 223.5±5.4 nm to 375.3±6.0 nm in 60days at room temperature, while at refrigerator condition it increased from 225.9±2.7 to 300.6±3.6 nm. For spray dried nanosuspension, particle size increased from 229.6±4.0 nm to 269.3±2.7 nm in 60days at room temperature (RT), while at refrigerator condition (RF) it increased from 226.9±4.1 to 251.1±3.6 nm. The crystal growth could be explained by Ostwald ripening. Generally, Ostwald ripening is a result of the difference in solubility between small and large particles due to a higher degree of curvature of small particles leading to higher solubility compared with the larger ones [34]. Thus, the SDCN showed lesser increase in particle size under both conditions compared to NS before spray drying. Thus, it is recommended to store the spray dried nanosuspension under refrigerator condition to minimize increase in particle size during storage.

#### Chemical stability

Assay results of CC in the NS and SDCN after 15, 30 and 60 days stored at room temperature (RT) and refrigeration conditions (RF) were shown in Table 1. The amount of CC remaining in the formulation was mainly dependent on type of surfactant and the storage condition. The percentage of active measured in case of NS was higher than SDCN.

The chemical stability observed was satisfactory at both conditions for 2 months as active content was more than 95% in all cases. Although the active contents are above 95%, it follows a downward trend with time so its storage should be maintained at refrigerated condition. Thus, it can be concluded that the chemical stability of CC was not affected due to milling and the formulation was stable for 2 months. Further studies are needed to prove long term stability of the formulation and to find out reasons for the downward trend. However, similar results are reported in some cases [35].

### In vivo study

As shown in Table 2, systolic blood pressure of UNX rats after 4 weeks was 135.0±5.2 mmHg compared to DOCA-salt rats 165.0±4.6 mmHg, i.e. only uninephrectomy was not sufficient to develop significant rise in systolic blood pressure. All DOCA-salt rats showed mild but significant hypertension in two weeks. Table 2 shows % decrease in systolic blood pressure of rats after one week of treatment.

**Fig. 7.**
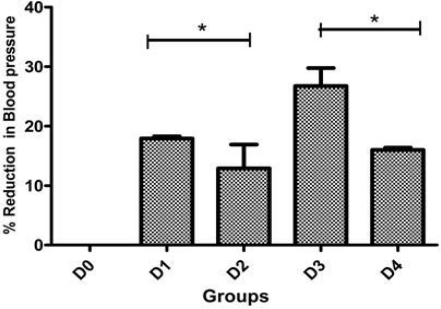
% Decrease in systolic blood pressure after treatment in different groups. (*…Indicates significant difference between two groups)

After one week treatment, D1 rats [receiving low dose nanosuspension] showed 17.94±0.36% and D2 rats [receiving low dose plain drug suspension] showed 12.89±0.42% decrease in systolic blood pressure. Similarly, D3 rats [receiving high dose nanosuspension] showed 26.75±0.34% and D4 rats [receiving high dose plain drug suspension] showed 16.0±0.38% decrease in systolic blood pressure. This significant enhancement in antihypertensive activity was clearly observed in Fig. 7 and attributed to nanosizing of candesartan cilexetil.

Thus, it was confirmed that Candesartan cilexetil decreases blood pressure in a dose-dependent manner and hence decrease in pressor effect can be directly correlated with the amount of drug that reaches systemic circulation i.e. bioavailability of drug. In other words, higher the inhibition of pressor effect, more the bioavailability of CC from administered formulation. The data also clearly demonstrated that inhibition of pressor effect was greater in rats receiving NS in comparison to rats receiving plain drug suspension at both doses. Based on this pharmacodynamic study, it could be concluded that bioavailability of CC was higher from NS in comparison to plain drug suspension, although it was difficult to quantify actual increase in bioavailability from this pharmacodynamic study.

## Conclusion

Formulation strategy of using nanocrystalline drug was investigated as a way to improve bioavailability of the Candesartan cilexetil. Media milling method using zirconium oxide beads was effective in nanosizing the drug and the spray dried nanosuspension showed improved stability compared to nanosuspension. XRD pattern and DSC results revealed the unchanged crystalline nature of CC in the spray dried product. Saturation solubility of CC was increased 22.44 times than that of bulk drug by nanosuspension formulation. In case of spray dried nanosuspension the increased surface area, higher saturation solubility and reduced diffusion distance led to increased dissolution velocity. The spray dried formulation was stable for two months at refrigeration condition. The results of the pharmacodynamic study showed significant reduction in blood pressure for nanosuspension as compare to plain drug suspension. Thus, the results of the *in vitro* and pharmacodynamic studies conclusively demonstrated significant enhancement in antihypertensive activity of candesartan when formulated as nanosuspension.

## Figures and Tables

**Tab. 1.** Assay of optimized batches of nanosuspension at various conditions

**Stability condition**	**Formulation**	**%Assay (±S.D.)**			

		**Initial**	**15days**	**30days**	**60days**
Room temperature	Nanosuspension	99.73±0.36	99.01±0.36	98.25±0.65	97.96±0.25

	Spray-dried Nanosuspension	98.75±0.35	98.05±0.36	97.59±0.85	96.98±0.25

Refrigerator	Nanosuspension	99.79±0.91	99.25±0.12	98.75±0.15	98.02±0.12

	Spray-dried Nanosuspension	98.69±0.61	98.14±0.16	97.75±0.35	97.18±0.14

**Tab. 2.** Mean systolic blood pressure in different groups of rats

**Group**		**Systolic blood pressure(mmHg)**					% Decrease in systolic blood pressure after treatment**

		**0 d**	**7 d**	**14 d**	**17 d**	**21 d**	
UNX rats		107±3.2	119±2.5	124±2.5	–	135±5.2	–

DOCA rats	D0	110±4.6	126±2.9	153±6.1	–	165±4.6	–
	D1	105±6.2	121±1.9	156±3.1	139±5.2	128±2.4	17.94±0.357^a^
	D2	109±3.9	124±3.8	152±5.9	142±3.2	138±3.2	12.89±0.425
	D3	115±5.4	129±5.2	157±5.9	127±5.2	115±2.1	26.75±0.335^a^
	D4	114±3.5	128±2.5	150±6.1	136±4.3	126±2.6	16.0±0.379

** %decrease in systolic blood pressure after one week of treatment in comparison to that of after 14 days (Actual measurements were done daily in all treatment groups but here for comparison one week time point was used); Each value represents the mean ± S.E.M. (n=4);

a *P* < 0.05, compared with rats receiving plain drug suspension; D0: DOCA control (no treatment); D1: rats receiving low dose nanosuspension; D2: rats receiving low dose plain drug suspension; D3: rats receiving high dose nanosuspension; D4: rats receiving low dose plain drug suspension.
